# The referral-to-attendance gap in vestibular rehabilitation: a retrospective cohort study in diverse South Florida patients

**DOI:** 10.3389/fneur.2026.1862003

**Published:** 2026-06-16

**Authors:** Madison Hawthorne, Luis Rodriguez-Diaz, Katie Miller, Kayla Minesinger, Devin Kennedy, Michael E. Hoffer, Erin Williams

**Affiliations:** 1 Department of Otolaryngology, University of Miami Miller School of Medicine, Miami, FL, United States; 2 Department of Neurological Surgery, University of Miami Miller School of Medicine, Miami, FL, United States; 3 Department of Biomedical Engineering, University of Miami, Miami, FL, United States

**Keywords:** Dizziness Handicap Index (DHI), Functional Gait Assessment (FGA), healthcare utilization, South Florida, vestibular disorders, vestibular rehabilitation therapy (VRT)

## Abstract

**Introduction:**

Despite strong evidence for vestibular rehabilitation therapy (VRT), the proportion of referred patients who actually attend remains poorly characterized. This study quantified the VRT referral-to-attendance gap in a South Florida cohort and evaluated whether sociodemographic and insurance-related factors were associated with treatment uptake and clinical outcomes measured by the Dizziness Handicap Index (DHI) and Functional Gait Assessment (FGA).

**Methods:**

We conducted a retrospective chart review of 243 adults aged 18–80 (observed range 25–79) who were recommended VRT at the University of Miami Ear Institute between 2012 and 2022. Demographic information (age, sex, race, ethnicity, insurance status), vestibular diagnoses, and outcome measures were extracted. Functional outcomes were assessed with baseline and follow-up DHI and FGA scores. Neighborhood-level socioeconomic status was characterized using the Area Deprivation Index (ADI). Disparities in VRT attendance and outcomes were evaluated using Chi-square tests, Firth’s penalized logistic regression, and analysis of covariance.

**Results:**

Of 243 patients referred for VRT, only 41% (*n* = 99) attended therapy, revealing a 59% referral-to-attendance gap, with a median of 7 visits (IQR 3–15) among attenders. No demographic, socioeconomic, insurance, or time variables were significantly associated with VRT attendance in unadjusted or multivariable analyses (all *p* > 0.05). In exploratory analyses limited to 25 patients with paired DHI data, DHI scores decreased by 15.2 points (95% CI −24.5 to −5.9; *p* = 0.002), though notably this did not exceed the 18-point minimum clinically important difference (MCID). Neighborhood deprivation (ADI) and insurance category were associated with DHI responder status. No medium-deprivation or publicly insured patients achieved the MCID, though no high-deprivation patients had paired data.

**Conclusion:**

No measured sociodemographic or clinical variable explained non-attendance, suggesting that structural or logistical barriers may be the primary drivers of this gap. Neighborhood deprivation and insurance category were associated with clinically meaningful DHI improvement in exploratory analyses, but these findings are constrained by a small, racially homogeneous analytic sample and require confirmation in larger prospective studies with standardized outcome collection.

## 1 Introduction

Vestibular dysfunction affects an estimated 35.4% of adults in the United States, with higher prevalence associated with increased age and other comorbidities including hypertension, diabetes, and tobacco use (1). Symptoms such as dizziness, imbalance, and spatial disorientation substantially impair patients’ functional capacity and are associated with declines in physical, mental, and social well-being (2). These individuals face up to a 12-fold increased risk of falls compared to those without vestibular impairment (1). Vestibular dysfunction also contributes to substantial healthcare system burden and associated economic costs, including emergency room visits and fall-related hospitalizations, particularly in aging populations (3).

Vestibular rehabilitation therapy (VRT) is an evidence-based, exercise-driven intervention designed to mitigate symptoms of vestibular dysfunction (4, 5). VRT is thought to promote central compensation and symptomatic improvement through targeted exercises that drive vestibular adaptation, habituation, and sensory substitution (2, 6). These processes may involve neuroplastic recalibration and adaptive sensory reweighting of vestibulo-ocular and vestibulo-spinal pathways (2, 6). Commonly prescribed for conditions including Meniere’s disease (MD), vestibular neuritis (VN), vestibular schwannoma (VS), mild traumatic brain injury (mTBI), benign paroxysmal positional vertigo (BPPV), and vestibular migraine (VM), VRT is widely regarded as a safe and effective intervention for patients (4, 5, 7–9).

Robust evidence supports VRT’s effectiveness in improving functional outcomes and reducing fall risk (10–13). Treatment success is commonly evaluated using clinically validated patient-reported outcomes capturing symptom burden and functional limitations. Prior studies have demonstrated substantial improvements in patient-reported outcomes, with both Dizziness Handicap Index (DHI) and Vestibular Disorders Activities of Daily Living (VADL) scores improving by 28 and 32%, respectively, following VRT in patients with both peripheral and central vestibular disorders (14). Performance-based outcomes also improve after VRT; Wellons et al. (15) demonstrated significant gains in Functional Gait Assessment (FGA) scores following outpatient vestibular rehabilitation. These improvements reflect not only symptom reduction but also enhanced confidence, mobility, and performance of daily activities.

Despite the high prevalence of vestibular dysfunction and the demonstrated benefits of VRT, a substantial gap persists between therapeutic demand and real-world implementation of this interventional treatment. A recent study reported that 68.8% of patients with vestibular neuritis or labyrinthitis initiated VRT after referral (16); however, broader data describing attendance rates among patients referred for VRT across vestibular diagnoses remain scarce. Current studies highlight several potential barriers contributing to this gap, including limited access to trained vestibular therapists, lack of awareness among providers and patients, financial constraints, and sociocultural factors such as mistrust and low health literacy (16–18). These barriers underscore the need to characterize real-world patterns of VRT delivery and identify patient- and system-level factors affecting access.

The primary aim of this retrospective chart review was to quantify the referral-to-attendance gap for VRT in a South Florida cohort and to characterize sociodemographic, insurance-related, and clinical factors associated with non-attendance. As a secondary aim, we evaluated whether these same factors were associated with clinically meaningful improvements in patients who did attend, as measured by the DHI and FGA. This secondary aim was completed in a subsample of patients who had both baseline and follow-up DHI or FGA values recorded.

## 2 Methods

The Institutional Review Board (IRB) at the University of Miami Miller School of Medicine granted permission for the conduction of this research (IRB #20230698) prior to the onset of data collection.

### 2.1 Study design

For this retrospective study, medical records were reviewed for patients prescribed VRT at the University of Miami Miller School of Medicine between 2012 and 2022. Inclusion criteria were: (1) adults aged 18–80 years, (2) diagnosed with a vestibular disorder including traumatic brain injury, vestibular schwannoma, Meniere’s disease, vestibular neuritis, vestibular migraine, benign paroxysmal positional vertigo (BPPV), sudden sensorineural hearing loss (SSNHL), vertigo, and/or dizziness, and (3) prescribed VRT between 2012 and 2022. Given the retrospective design, no *a priori* power analysis was performed, and the sample size (*n* = 243) was determined by the number of eligible patients meeting inclusion criteria. Pertinent clinical and demographic data were abstracted for each patient. Clinical variables included vestibular diagnosis, timelines from symptom onset to referral, number of VRT visits attended, and clinical improvement.

Demographic variables included age, sex, marital status, race, ethnicity, zip code, and insurance plan. Neighborhood-level socioeconomic status (SES) was characterized using the Area Deprivation Index (ADI), a validated composite measure incorporating 17 census indicators of income, education, employment, and housing quality at the block-group level (19). Patient ZIP codes were linked to the 2023 ADI (v4.0.1) for Florida; the median national ADI percentile across all block groups within each patient’s 5-digit ZIP code was assigned. ADI national rankings are reported as percentiles from 1 to 100, with higher ADI percentiles indicating greater neighborhood deprivation (20). ADI-based SES was thus categorized into tertiles to facilitate interpretation: low deprivation (national percentile 1–33), medium deprivation (34–66), or high deprivation (67–100).

Improvement was defined by the minimum clinically important difference (MCID) scores for the DHI (≥18 points) and FGA (≥4 points) (15). The DHI is a patient-reported questionnaire consisting of 25 items categorized into physical, emotional, or functional subscales, with responses scored as yes (4), maybe (2), or no (0), yielding a total score ranging from 0 to 100. Scores from 0 to 30 are classified as mild, 31–60 as moderate, and 61–100 as severe disability (19). The FGA is a 10-item test performed by health professionals to assess patients’ postural stability and their ability to perform multiple motor tasks while walking. Each item is scored as severe impairment (0), moderate impairment (1), mild impairment (2), or normal ambulation (3), with scores ranging from 0 to 30, with a lower score indicating more severe impairment (15). Among patients with more than one recorded DHI or FGA assessment, the scores nearest to therapy onset were selected as the baseline treatment measure, and the latest available follow-up scores, furthest from therapy onset, were used to calculate change.

**Figure 1:**
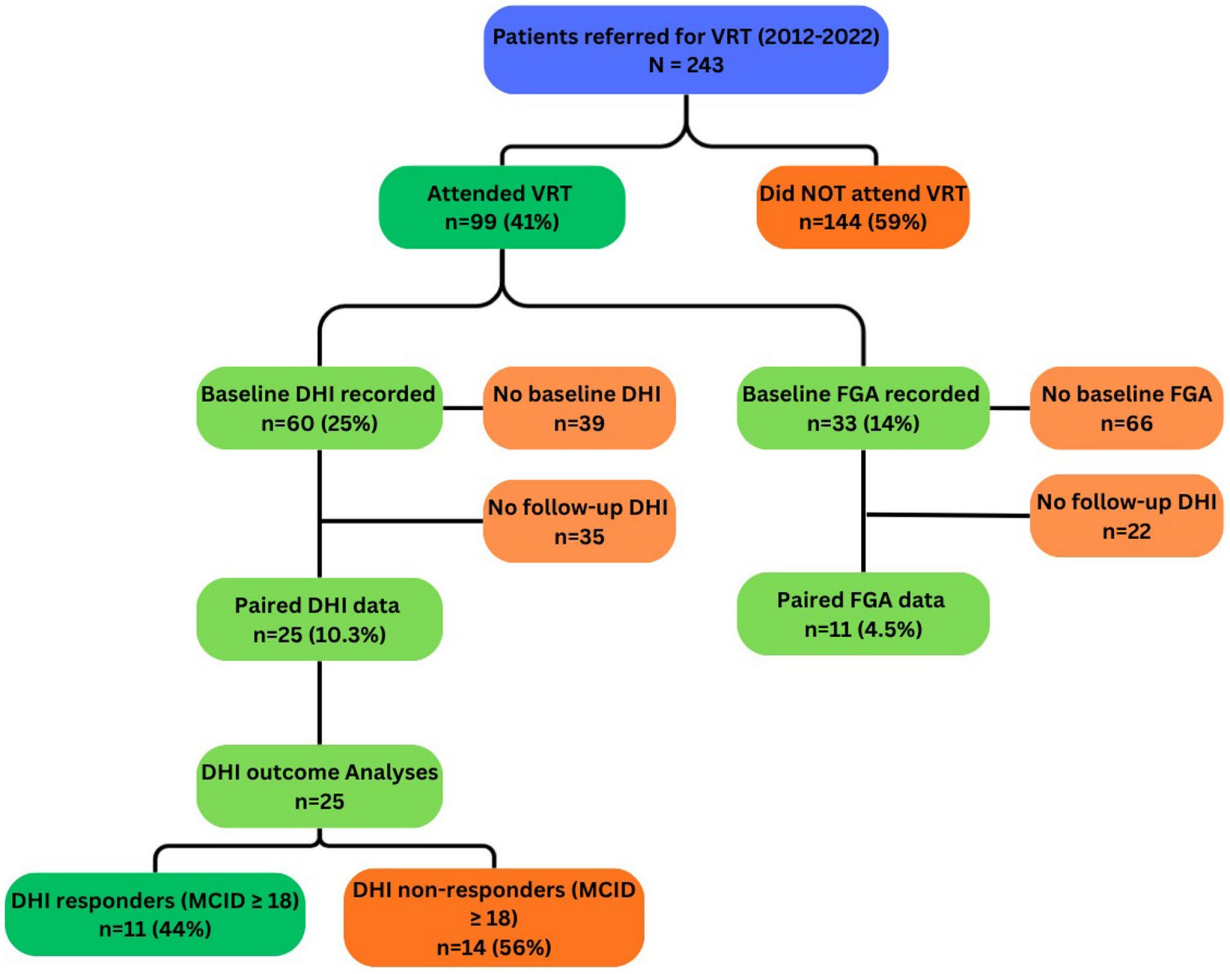
STROBE participant flow diagram. Attrition cascade from 243 patients referred for VRT to 25 with paired DHI data used in outcome analyses. Of the 99 patients (41%) who attended VRT, 60 had baseline DHI scores recorded, and 25 had both baseline and follow-up DHI assessments. Patients with paired data had significantly higher baseline DHI (58.8 vs. 42.2, *p* = 0.015) and differed in diagnostic distribution (*p* = 0.034) compared with those without paired data. Eleven of 25 patients (44.0%) achieved clinically meaningful improvement (MCID ≥18 points).

As a secondary measure, SES was also categorized by median household income (MHI) from the U. S. Census Bureau American Community Survey (2012–2022): low (<$55,000), middle ($55,000–$85,000), or high (≥$85,000). Insurance plans were further classified by insurance type (commercial, exchange/marketplace, Medicaid, Medicare, Medicare Advantage, Military/Tricare, and Worker’s Comp) and category, defined as public or private. Exchange/Marketplace plans were classified as “public” in the analytic variable, consistent with their subsidized enrollment structure. Data collection and organization are outlined in Figure 1.

### 2.2 Statistical analysis

Descriptive statistics summarized demographic and clinical characteristics. Statistical significance was defined as a two-sided *p* < 0.05, and all analyses used complete-case analysis with R (version 4.4.1; R Foundation for Statistical Computing).

#### 2.2.1 Attendance analyses

VRT attendance was defined as completion of at least one documented vestibular rehabilitation visit following referral. Patients with no documented VRT visits after referral were classified as non-attenders. Associations between sociodemographic variables and VRT attendance were evaluated using Chi-square tests (Fisher’s exact when expected cell counts were small), with Cramer’s V and bootstrap 95% CIs (2,000 replicates) reported as effect sizes. Cramer’s V was interpreted using conventional benchmarks, with values of approximately 0.10, 0.30, and 0.50 considered small, medium, and large effects for 2 × 2 comparisons, respectively; for larger contingency tables, these thresholds were interpreted cautiously because effect-size magnitude depends on table dimensions. Multivariable logistic regression was also performed for VRT attendance, including age, sex, race, insurance category, and ADI percentile as simultaneous predictors. Because publicly insured patients were substantially older than privately insured patients (mean 67 vs. 55 years), nearest-neighbor age matching was performed as a sensitivity analysis to balance this clinically plausible confounder between insurance groups. Matching was conducted at a 1:1 ratio with a 0.25 SD caliper without replacement using the MatchIt package in R. Although age was not independently associated with VRT attendance, this approach allowed comparison of attendance rates after accounting for the age imbalance.

#### 2.2.2 Outcome analyses

Paired baseline and follow-up differences in DHI and FGA scores were evaluated using Wilcoxon signed-rank tests, with rank-biserial correlation (r) as the effect size. Effect sizes were interpreted using conventional thresholds, recognizing that these benchmarks are heuristic: for rank-biserial correlation, r values of approximately 0.10, 0.30, and 0.50 were considered small, medium, and large effects, respectively; for partial η^2^, values of approximately 0.01, 0.06, and 0.14 were considered small, medium, and large effects, respectively (21). Due to limited paired FGA data (*n* = 11 with both baseline and follow-up scores), outcome analyses focused primarily on the DHI (*n* = 25). Predictors of follow-up DHI were assessed using ANCOVA with follow-up DHI as the dependent variable and baseline DHI as a covariate (Type III sums of squares; partial η^2^ reported). Covariates tested in separate models included ADI, MHI-based SES, insurance category, diagnosis, sex, age, and number of visits. To adjust for potential selection bias, baseline characteristics of patients with and without paired outcome data were compared using Wilcoxon rank-sum and Fisher’s exact tests. Hodges-Lehmann pseudomedian differences with 95% confidence intervals from the Wilcoxon signed-rank test are reported for paired comparisons, ensuring consistency between the confidence interval and the hypothesis test. ANCOVA model assumptions were assessed using the Shapiro–Wilk test for residual normality and Levene’s test for homogeneity of variance. Omega-squared was reported alongside partial eta-squared as a less biased effect size estimate for small samples (21).

#### 2.2.3 Responder analyses

Clinically meaningful improvement was defined as achievement of the MCID (DHI ≥ 18-point decrease; FGA ≥ 4-point increase) (15, 22). Associations between responder status and sociodemographic factors were evaluated using Fisher’s exact tests. Because complete separation occurred in several models (zero responders in certain subgroups), Firth’s penalized logistic regression was used to obtain finite odds ratio estimates with profile-penalized likelihood confidence intervals. Given the number of predictors tested across multiple analytic frameworks, all outcome and responder analyses should be considered exploratory and hypothesis-generating; no correction for multiple comparisons was applied. To assess potential informative missingness, the probability of having paired DHI data among VRT attenders was compared across ADI, insurance, and demographic categories using nonparametric Fisher’s exact tests.

## 3 Results

### 3.1 Demographics

Our final cohort included 243 patients (mean age 57 ± 12.4 years; range 25–79; no patients younger than 25 met inclusion criteria during the study period; inclusion criteria specified ages 18–80), of whom 168 (69%) were female (Table 1). Most patients identified as White (*n* = 198, 81%), followed by Black/African American (*n* = 22, 9%); nearly half identified as Hispanic/Latino (*n* = 115, 47%).

**Table 1:** Demographic description of the study population.

	Attender % (*N* = 99)	Non-attender % (*N* = 144)	Total Cohort % (*N* = 243)
Avg. age (years)	58	57	57
Sex			
Male	14% (35)	16% (40)	31% (75)
Female	26% (64)	43% (104)	69% (168)
Race			
White	33% (81)	48% (117)	81% (198)
Black/AA	5% (13)	4% (9)	9% (22)
Asian	0	0.4% (1)	0.5% (1)
Am. Indian/AK Native	0	0.4% (1)	1% (2)
Multiple	0	0.4% (1)	0.5% (1)
Unknown	2% (5)	6% (14)	8% (19)
Ethnicity			
Hispanic/Latino	22% (54)	25% (61)	47% (115)
Non-Hispanic/Latino	29% (70)	16% (40)	45% (110)
Unknown	2% (5)	5% (13)	8% (18)

Patient demographics, including sex, race, and ethnicity, organized by VRT attendance (y/n). Most patients identified as White and Hispanic/Latino.

Compared with Miami-Dade County, where approximately 70% of residents identify as Hispanic/Latino and 16% as Black/African American (23), our cohort included a lower proportion of Hispanic/Latino patients (47%) and Black/African American patients (9%). The cohort was also older and more female than the county population.

Under the traditional classification, 201 patients (83%) had private insurance and 42 (17%) had public insurance. In the analytic classification used for all inferential analyses, Exchange/Marketplace plans were reclassified as public, yielding 170 (70%) private and 73 (30%) public (see Methods). Publicly insured patients were older than those with private insurance (mean 64 vs. 54 years). The median neighborhood ADI national percentile was 35 (IQR 22–45); of the 234 patients with matched ADI data, 104 (44%) resided in low-deprivation, 118 (50%) in medium-deprivation, and 12 (5%) in high-deprivation neighborhoods.

The most common vestibular diagnoses were Meniere’s disease (*n* = 94, 39%), vestibular neuritis (*n* = 79, 33%), and vestibular schwannoma (*n* = 41, 17%), with smaller proportions diagnosed with vestibular migraine (*n* = 8, 3%), mTBI (*n* = 7, 3%), and BPPV (*n* = 4, 2%). An additional 27 patients (11%) were classified as “other,” which included less frequent diagnoses like SSNHL, vertigo, and dizziness. Because some patients had multiple diagnoses, categories were not mutually exclusive; 10 patients (4%) appeared in more than one category (Supplementary Table 1).

### 3.2 Vestibular rehabilitation attendance

Among the cohort of 243 patients referred for VRT, 59% (*n* = 144) did not attend therapy, representing a substantial referral-to-attendance gap. The remaining 41% (*n* = 99) attended at least one session, with a median of 7 visits (IQR 3–15) among attenders. The median time from VRT recommendation to the patient’s first visit was 93 days (IQR 17.2–331.5). No sociodemographic or clinical variable was significantly associated with VRT attendance (all *p* > 0.05; Table 2). Effect sizes were uniformly small (Cramer’s V = 0.03–0.21). Supplementary Figure 1 depicts VRT attendance by diagnosis.

**Table 2:** VRT attendance predictors (*N* = 243).

Predictor	Test statistic	*p* value	Effect size
Sex	χ^2^ (1) = 1.24	0.265	V = 0.07 [0.00–0.20]
Race	χ^2^ (5) = 7.46	0.189	V = 0.18 [0.10–0.29]
Ethnicity	χ^2^ (2) = 3.97	0.138	V = 0.13 [0.04–0.25]
Insurance category	χ^2^ (1) = 0.25	0.616	V = 0.03 [0.00–0.19]
ADI category	χ^2^ (2) = 3.50	0.174	V = 0.12 [0.04–0.24]
SES (MHI)	χ^2^ (2) = 0.86	0.650	V = 0.06 [0.02–0.20]
Diagnosis	χ^2^ (9) = 10.82	0.288	V = 0.21 [0.18–0.37]
Age (per year)	OR = 1.00 [0.98–1.02]	0.871	—
Insurance (age-matched)	OR = 2.05 [0.82–5.27]	0.139	—

Summary of associations between sociodemographic and clinical variables and VRT attendance. Cramer’s V with bootstrap 95% CIs quantifies effect sizes. No predictor reached significance.

**Figure 2:**
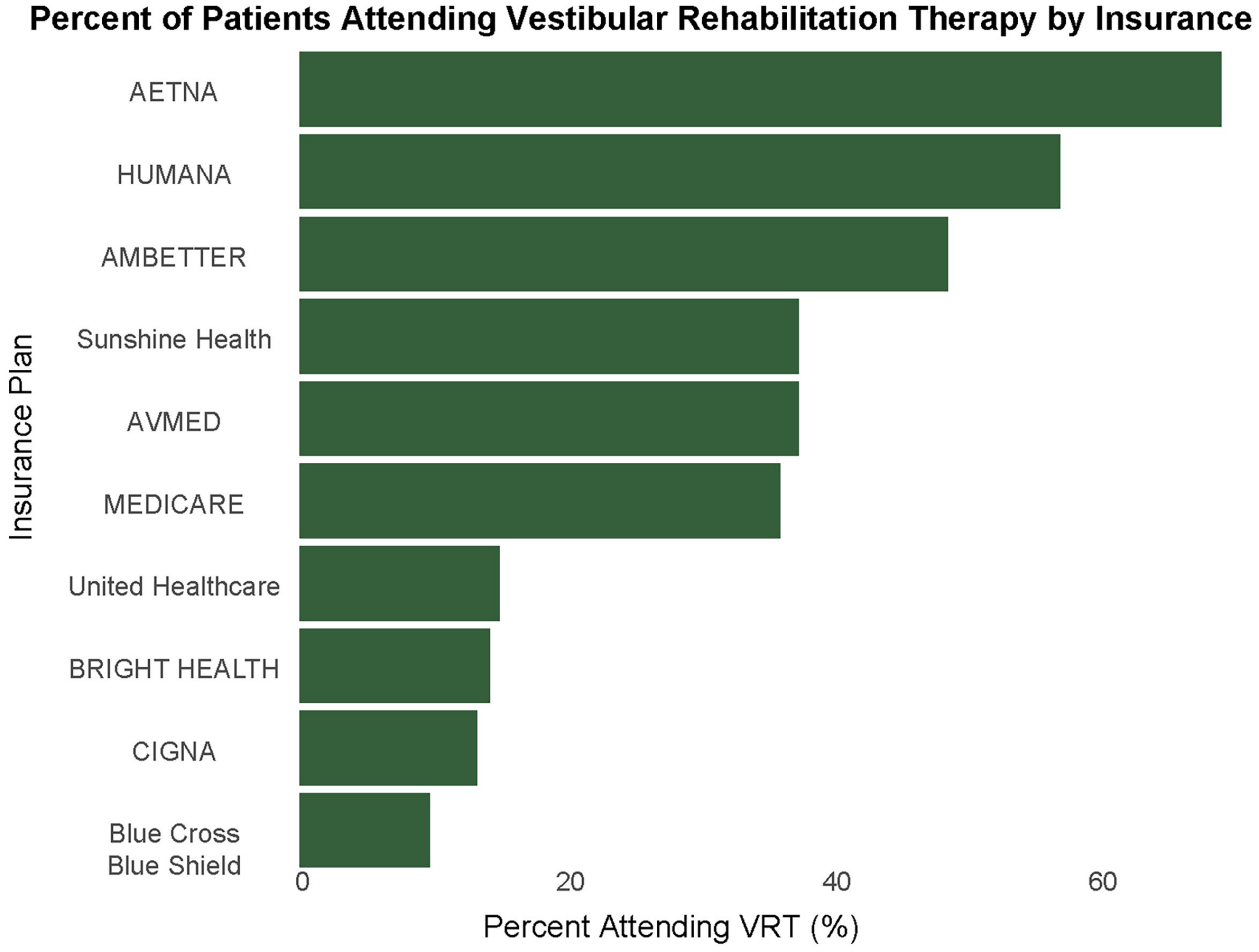
VRT attendance by insurance plan. Percentage of patients attending VRT by insurance plan, ordered from highest to lowest attendance rate. Only the ten most common plans are shown; all 28 plans were included in statistical analyses. Plan-level attendance varied significantly (χ^2^ (27) = 59.4, *p* < 0.001), though broader insurance category (public vs. private) was not significant (*p* = 0.62).

VRT attendance varied across individual insurance plans (Figure 2). Blue Cross Blue Shield, CIGNA, Medicare, Oscar Health Exchange, and United Healthcare had lower attendance rates compared with the reference group (Aetna). Because 28 distinct insurance plans were recorded (df = 27), resulting in small cell counts for many plans, comparisons at the individual plan level were considered descriptive. Formal statistical analyses were therefore performed using broader insurance classifications; insurance type and insurance category were not significantly associated with VRT attendance (insurance type: *p* = 0.41; insurance category: *p* = 0.62, respectively).

Logistic regression analyses corroborated these findings (Table 2). Demographic, diagnostic, and insurance variables were not significantly associated with VRT attendance. After matching public and private insurance patients on age (*n* = 50 per group; mean age 60.7 vs. 60.1 years), publicly insured patients demonstrated higher attendance rates (42% vs. 26%), though this difference was not statistically significant (Fisher’s exact *p* = 0.14; OR 2.05, 95% CI 0.82–5.27).

*Multivariable Attendance Model.* In multivariable logistic regression (*N* = 243, AIC = 329.73), insurance category (OR 1.58, 95% CI 0.84–2.98, *p* = 0.159), age (OR 0.99, *p* = 0.454), sex (OR 1.58 for male, *p* = 0.120), and race were not independently significant, though Black/African American race showed a trend toward higher attendance (OR 2.31, 95% CI 0.92–5.78, *p* = 0.074). Adding continuous ADI to the model (*n* = 234) did not improve fit (LRT *p* = 0.542).

### 3.3 Vestibular rehabilitation outcomes

VRT outcomes were examined using available DHI and FGA scores recorded before and after VRT. Baseline treatment scores were defined as the recorded assessment closest to therapy onset, and follow-up scores were defined as the latest available follow-up assessment after therapy initiation; therefore, paired outcomes reflected available clinical documentation rather than standardized collection exclusively at initial evaluation and discharge. Given the limited paired outcome sample and informative missingness described below, all outcome and responder analyses were considered exploratory and should be interpreted as hypothesis-generating. Of the 243 patients, 60 (~25%) had a baseline DHI and 33 (~14%) had a baseline FGA recorded. Mean baseline DHI was 49.6 ± 24.9, representing moderate disability; mean baseline FGA was 22.6 ± 5.6, representing mild disability (Table 3). Improvement could only be assessed among patients with both baseline and follow-up measurements; 25 patients had paired DHI data and 11 had paired FGA data (Table 3). Patients with paired DHI data had significantly higher baseline DHI scores than those without (58.8 vs. 42.2, *p* = 0.015) and differed in diagnostic distribution (Fisher’s exact *p* = 0.034), but did not differ in age, sex, race, insurance category, or SES (all *p* > 0.15). Given the limited paired FGA sample, outcome analyses are focused primarily on the DHI.

**Table 3:** Summary of VRT outcome data.

Outcome measure	Baseline (Mean ± SD)	Paired baseline VRT (Mean ± SD)	Paired follow-up VRT (Mean ± SD)	Mean difference (95% CI)
DHI	49.6 ± 24.9 (*n* = 62)	58.8 ± 25.1 (*n* = 25)	43.6 ± 29.0 (*n* = 25)	−15.2 (−24.5 to −5.9)
FGA	22.6 ± 5.6 (*n* = 33)	20.6 ± 6.3 (*n* = 11)	25.8 ± 4.9 (*n* = 11)	−5.5 (−11 to 1)

Baseline scores are reported for all patients with available pre-VRT data. Changes were evaluated in the subset with paired measurements. Paired baseline VRT means differ from overall baseline means because they reflect only patients who also had follow-up VRT scores. DHI decreased by a mean of 15.2 points (Wilcoxon V = 239.5, *p* = 0.002). FGA did not significantly change (V = 10.5, *p* = 0.092). MCID is ≥18 points for the DHI and ≥4 points for the FGA.

**Figure 3:**
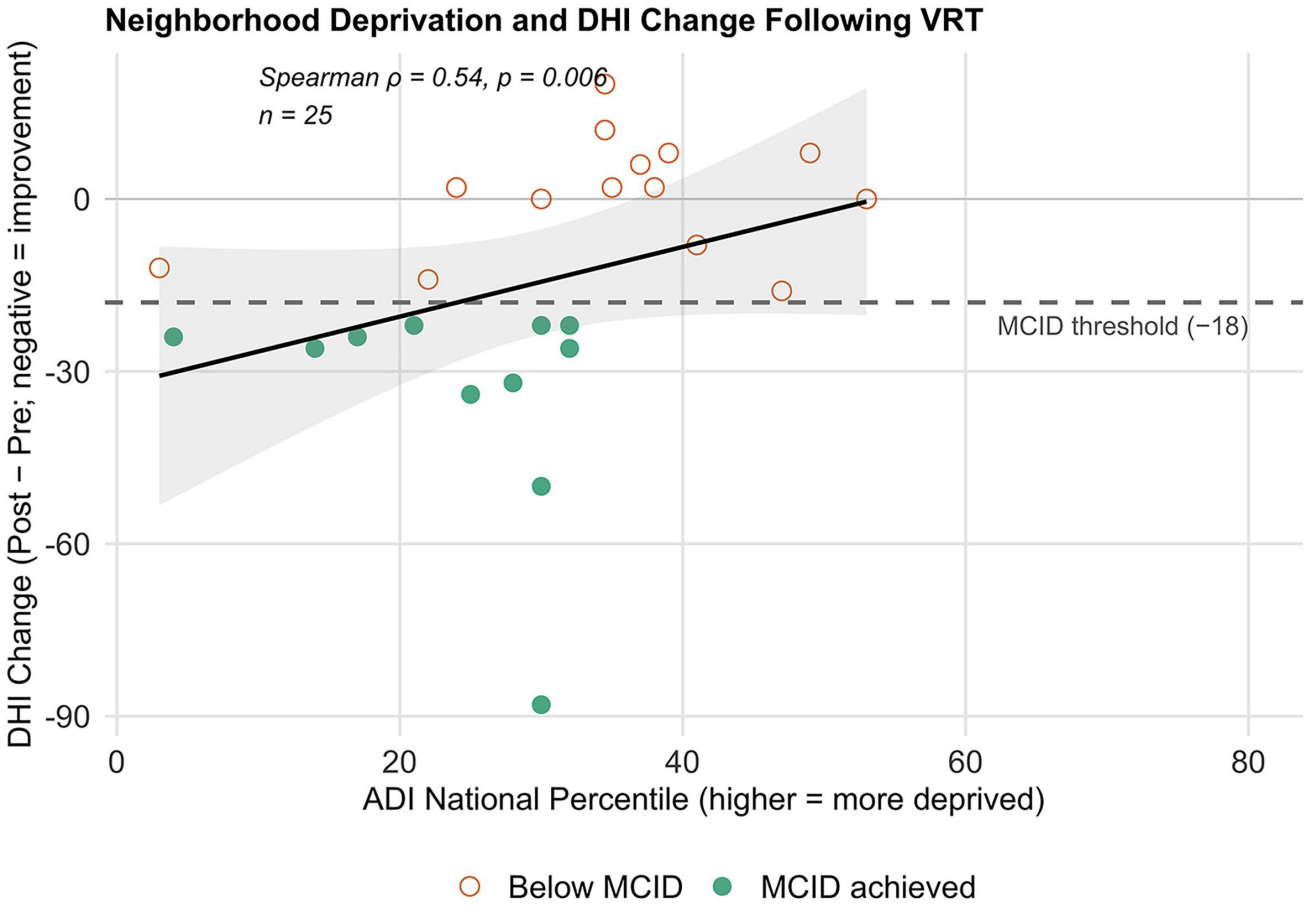
Neighborhood deprivation and DHI change following VRT. Scatter plot of ADI national percentile (*x*-axis; higher = more deprived) versus DHI change (*y*-axis; negative = improvement) for patients with paired DHI data (*n* = 25 with ADI data). Dashed line indicates MCID threshold (−18 points). Filled circles represent patients achieving MCID; open circles represent non-responders. The linear trend (Spearman rho = 0.54, *p* = 0.006) indicates that patients in more deprived neighborhoods showed less improvement.

A Wilcoxon signed-rank test indicated a significant decrease in DHI scores following VRT (V = 239.5, *p* = 0.002, rank-biserial r = 0.615; Hodges-Lehmann pseudomedian 15, 95% CI 6–24). ADI score was correlated with DHI change (Spearman rho = 0.54, *p* = 0.006, n = 25); patients from low-deprivation neighborhoods had a mean DHI change of −26.3 (SD 21.3), while medium-deprivation patients had a mean change of +3.4 (SD 10.1) (Figure 3). Privately insured patients improved more (mean −17.0, SD 24.6) than publicly insured patients (mean −5.7, SD 9.4). ANCOVA models adjusting for baseline DHI (Table 4) were examined as sensitivity analyses, though residual non-normality in the ADI model (Shapiro–Wilk *p* = 0.010) limited parametric inference. ADI category (e.g., low deprivation, medium deprivation) was associated with follow-up DHI score (*F* (1, 21) = 21.56, *p* < 0.001, ω^2^ = 0.46; *n* = 24, comparing low- and medium-deprivation groups only), with low-deprivation patients showing greater improvement (mean DHI change −26.3 vs. −3.4 points for medium-deprivation patients). Given violation of the normality assumption in the ADI model, a permutation-based sensitivity analysis supported the parametric finding (permutation *p* < 0.0001). MHI-based SES was not significant in ANCOVA (*F* (2, 21) = 3.11, *p* = 0.065, ω^2^ = 0.14). Insurance category, diagnosis, visits, age, and sex were not significant in separate ANCOVA models (all *p* > 0.25).

**Table 4:** ANCOVA models predicting follow-up DHI.

Model	Predictor	Df	Sum of squares	Mean square	*F* value	*p* value	Partial η^2^
A	ADI category	1	5772.3	5772.3	21.56	<0.001	0.51
A	Baseline DHI	1	5672.0	5672.0	21.19	<0.001	0.50
A	Residuals	21	5621.7	267.7	—	—	—
B	MHI-based SES	2	2607.8	1303.9	3.11	0.065	0.23
B	Baseline DHI	1	4056.6	4056.6	9.69	0.005	0.32
B	Residuals	21	8792.4	418.7	—	—	—

Models use follow-up DHI as the dependent variable with baseline DHI as a covariate (Type III SS). Model A uses ADI-based neighborhood deprivation; Model B uses MHI-based SES categories. *N* = 24–25 for each model.

Clinically meaningful DHI improvement (≥18-point decrease) was examined descriptively across SES subgroups. All 11 responders resided in low-deprivation neighborhoods; no patients from medium- or high-deprivation areas achieved MCID (Fisher’s exact *p* < 0.001). A consistent pattern was observed across alternative SES measures: no low-MHI patients achieved MCID (Fisher’s exact *p* = 0.009), and no publicly insured patients achieved MCID compared with 55% of privately insured patients (Fisher’s exact *p* = 0.024). These findings were consistent with continuous ADI analyses (Figure 3). Given complete separation in several subgroups and the small analytic sample (*n* = 25), Firth’s penalized regression estimates are reported in Table 5 for transparency but should not be interpreted as stable effect size estimates. Race, ethnicity, sex, and diagnosis were not associated with responder status (all *p* > 0.30). All responder analyses are exploratory and hypothesis-generating.

**Table 5:** DHI responder status by SES and insurance category (*n* = 25).

Grouping variable	*n*	Non-responder	Responder	Responder rate
ADI category				
Low deprivation	15	4	11	73.3%
Medium deprivation	10	10	0	0%
High deprivation	0	—	—	—
MHI-based SES				
Low	8	8	0	0%
Middle	14	5	9	64.3%
High	4	2	2	50.0%
Insurance category				
Private	20	9	11	55.0%
Public	6	6	0	0%

Patients classified as responders (≥18-point DHI decrease) or non-responders. Complete separation is evident: no responders were observed in medium- or high-deprivation neighborhoods (ADI), in the low MHI group, or among publicly insured patients.

### 3.4 Differential follow-up analysis

Among the 99 VRT attenders, 25 (25.2%) had paired DHI data. The probability of having paired data did not differ significantly by insurance category (Private 28.4% vs. Public 18.8%, Fisher’s exact *p* = 0.336), ADI category (Fisher’s exact *p* = 0.164), or MHI-based SES (Fisher’s exact *p* = 0.161). However, race was significantly associated with paired data availability: all 25 patients with paired DHI data were White, while none of the 13 non-White attenders had paired data (Fisher’s exact *p* = 0.014), though notably 62 % (*n* = 16) of patients with paired DHI data identified as Hispanic or Latino. Further, patients with paired data tended to reside in less deprived neighborhoods (Wilcoxon *p* = 0.094; median ADI 31 vs. 38). Among attenders with baseline DHI scores (*n* = 60), those with follow-up scores had significantly higher baseline DHI (58.8 vs. 42.2, *p* = 0.015), confirming selection toward more symptomatic patients. These findings indicate that outcome analyses are subject to informative missingness, particularly by race.

### 3.5 VRT dose, follow-up interval, and baseline-follow-up correlation

Among all attenders, visits did not differ by insurance category (median 7 and 9 visits for private and public insurance, respectively; *p* = 0.747), though patients from low-deprivation neighborhoods attended more sessions (median 10.5 vs. 6.0; *p* = 0.037). Among the 25 patients with paired DHI data, publicly insured patients completed more visits (median 24 vs. 13). Among patients with paired DHI data and valid referral and treatment dates (*n* = 21), time from referral to first VRT visit (median 173 days) was not significantly associated with DHI responder status (Fisher’s exact *p* = 0.41). The median interval between baseline and follow-up DHI assessments was 184 days (6.0 months; IQR 80–465; range 21–2,321 days), with no significant difference by insurance (Private: 134 days; Public: 292 days; *p* = 0.514). The baseline-follow-up DHI correlation was r = 0.65 (Pearson), rho = 0.63 (Spearman).

## 4 Discussion

This retrospective cohort study of 243 patients referred for VRT at a single South Florida center documents a substantial referral-to-attendance gap: 59% of referred patients never attended therapy. This rate, which to our knowledge has rarely been reported across a broad range of vestibular diagnoses, underscores that referral alone is insufficient to ensure treatment uptake. Notably, no measured demographic, diagnostic, or socioeconomic variable significantly explained non-attendance, suggesting that unmeasured structural or logistical barriers may be primary drivers of the gap, though direct evidence for this in VRT non-attendance remains scarce. In exploratory outcome analyses limited to 25 patients with paired DHI data, neighborhood deprivation and insurance category were associated with responder status, though these findings are substantially constrained by the small sample, complete separation in several models, and evidence of informative missingness.

The 41% attendance rate is lower than the 68.8% reported by Lodha et al. (16) for vestibular neuritis and labyrinthitis, likely reflecting our broader diagnostic inclusion and differences in clinical settings. Attendance varied across individual insurance plans (Chi-squared *p* < 0.001) but was attenuated when collapsed into public versus private categories, suggesting within-group heterogeneity driven by plan-specific factors such as copayments, authorization requirements, and network availability (24, 25), though none were directly measured. Importantly, the 59% non-attendance rate observed here represents one of the few quantitative estimates of the referral-to-attendance gap for VRT across a broad spectrum of vestibular diagnoses. Although studies in adjacent rehabilitation fields report non-attendance rates of 30–50% for outpatient physical therapy (24, 25), comparable figures for VRT specifically are largely absent from the literature. Further, while prior work has documented structural barriers affecting vestibular rehabilitation outcomes (14) and barriers to adherence among enrolled patients (13), the specific drivers of the referral-to-attendance gap remain poorly characterized in the literature. Our finding that no measured sociodemographic variable explained non-attendance, combined with the 93-day median delay to first visit among attenders, is consistent with, though does not confirm, the presence of unmeasured logistical barriers such as scheduling, transportation, or cost. Prospective studies capturing these variables directly are needed, as simply increasing referrals without addressing access barriers is unlikely to close this gap.

Among patients with paired DHI data, scores decreased by a mean of 15.2 points (Hodges-Lehmann pseudomedian 15, 95% CI 6–24; *p* = 0.002), reflecting statistically significant subjective improvement in dizziness-related handicap. However, the average improvement did not exceed the 18-point MCID and therefore did not meet the conventional threshold for clinically meaningful change at the group level. In the smaller subset with paired FGA data (*n* = 11), scores did not significantly change between baseline and follow-up visits (Wilcoxon V = 10.5, pseudomedian −5.5, 95% CI − 11 to 1; *p* = 0.092). Given the small sample and non-significant result, FGA outcomes are not interpretable in this cohort. Regression-to-the-mean analyses suggest that the expected RTM effect is modest (~3.1 points at the observed baseline-follow-up correlation of r = 0.65), but without a control group, treatment effect cannot be separated from natural recovery. Neighborhood deprivation was associated with DHI change in non-parametric analysis (Spearman rho = 0.54, *p* = 0.006, *n* = 25); parametric ANCOVA elicited a directionally consistent result (ω^2^ = 0.46), though residuals violated normality (Shapiro–Wilk *p* = 0.010). However, the differential follow-up analysis revealed that all 25 patients with paired data were White (Fisher’s exact *p* = 0.016) and mostly Hispanic, and patients with paired data tended to reside in less deprived neighborhoods (Wilcoxon *p* = 0.094). The complete separation therefore likely reflects a combination of real disparities and differential measurement. The absence of publicly insured responders was not attributable to fewer treatment sessions, as publicly insured patients with paired data completed more visits (median 24 vs. 13).

Several limitations warrant consideration. Only 25 of 243 patients (10.3%) contributed to outcome analyses, all of whom were White, precluding any inference about racial disparities in VRT outcomes and limiting generalizability. This homogeneity may reflect broader patterns of healthcare access or documentation disparities, each of which merits further investigation. Patients with paired data had higher baseline DHI scores (58.8 vs. 42.2, *p* = 0.015). SES was characterized using ZIP code-linked ADI, an ecological measure that may not reflect individual circumstances. ANCOVA residuals in the ADI model were non-normal (Shapiro–Wilk *p* = 0.010), and partial η^2^ estimates (0.51) are likely inflated (ω^2^ = 0.46). Concordant findings across ADI, MHI, and insurance should not be interpreted as independent replication, as these are correlated SES proxies. The absence of a control group is a fundamental constraint; observed DHI and FGA changes cannot be attributed to VRT with confidence, as natural recovery, regression to the mean, and placebo effects cannot be ruled out. The 10-year study period (2012–2022) spans the ACA expansion and COVID-19 pandemic with no temporal adjustment. Although paired outcome data were reviewed to ensure that baseline and follow-up measurements corresponded to the same vestibular diagnosis or clinical indication, follow-up intervals varied widely (21–2,840 days; median 184 days) and outcome assessment timing was non-standardized. Therefore, these paired analyses should be interpreted as exploratory and may not fully reflect outcomes from a uniform episode of care. Additionally, language data, including preferred language, interpreter use, and acculturation level, were unavailable despite 47% Hispanic/Latino composition. These variables may independently influence VRT attendance and outcomes through pathways such as provider-patient communication barriers, health literacy, and cultural attitudes toward rehabilitation, thus representing a meaningful gap in our equity analysis. Lastly, complete separation produced confidence intervals spanning orders of magnitude, underscoring the need for larger prospective studies with standardized outcome collection.

## 5 Conclusion

This study documents a 59% referral-to-attendance gap for VRT across a broad range of vestibular diagnoses, a figure that, to our knowledge, has rarely been quantified. No measured sociodemographic, insurance, or clinical variable explained non-attendance, suggesting that structural and logistical barriers may be the primary drivers. Exploratory outcome analyses in 25 patients with paired DHI data suggested that neighborhood deprivation and insurance category may be associated with clinically meaningful improvement, though the small, racially homogeneous sample limits inference. These findings indicate that closing the gap requires interventions beyond the point of referral, and that prospective studies with standardized outcome collection and more diverse samples are needed to confirm these associations.

## Data Availability

The raw data supporting the conclusions of this article will be made available by the authors, without undue reservation.
