# Supplementary material for: The referral-to-attendance gap in vestibular rehabilitation: a retrospective cohort study in diverse South Florida patients
Source: Front Neurol. 2026 Jun 16;17:1862003. doi: 10.3389/fneur.2026.1862003 (PMC13314503; doi:10.3389/fneur.2026.1862003)
Supplement: Supplementary file 1 [file Supplementary_file_1.DOCX]

Supplementary Material

# Supplementary Figures


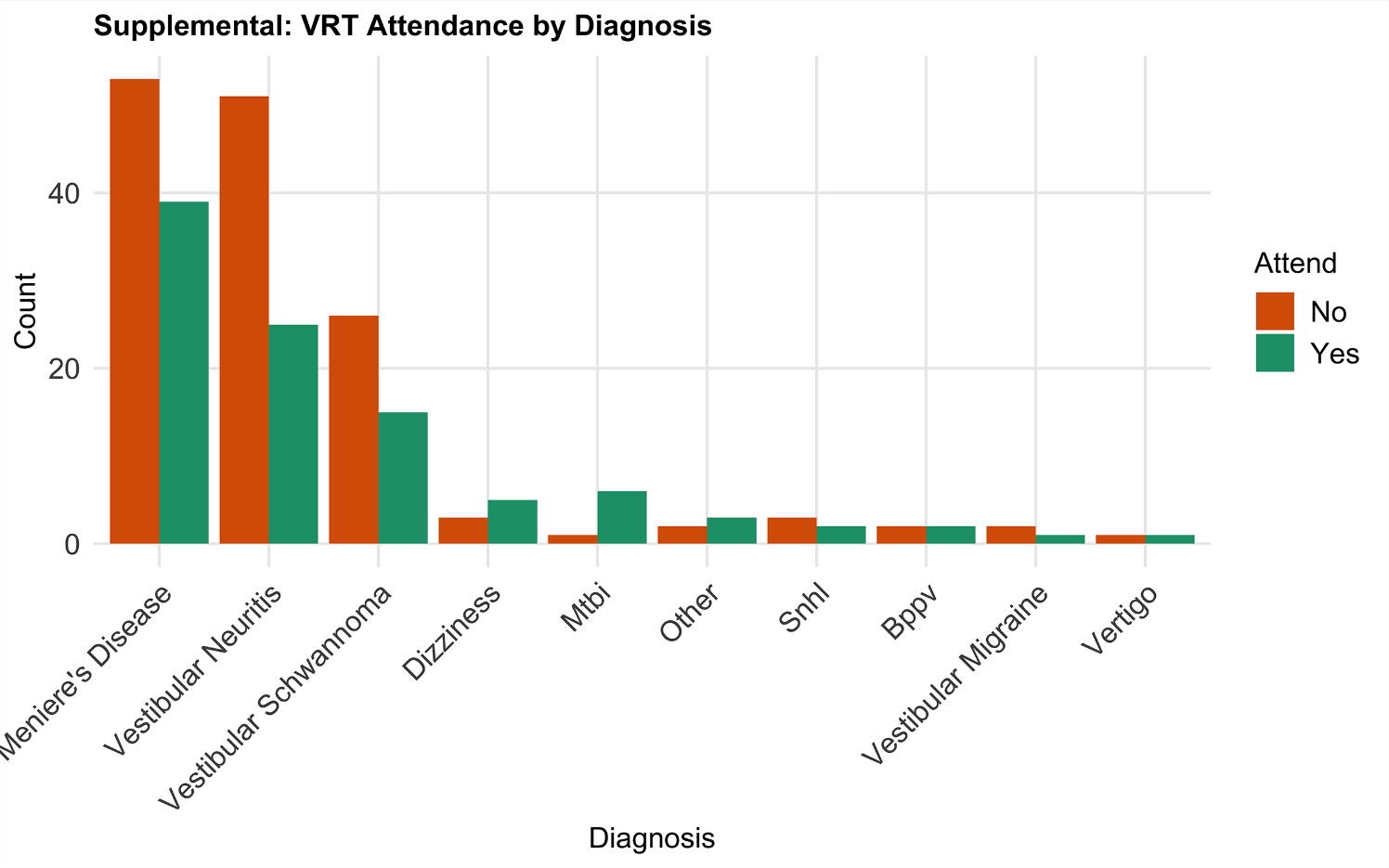


**Supplementary Figure 1. VRT Attendance by Vestibular Diagnosis.** Number of patients who attended versus did not attend VRT across diagnoses, ordered by decreasing attendance count. Diagnosis was not significantly associated with attendance (χ²(9) = 10.82, p = 0.288, Cramer’s V = 0.21).


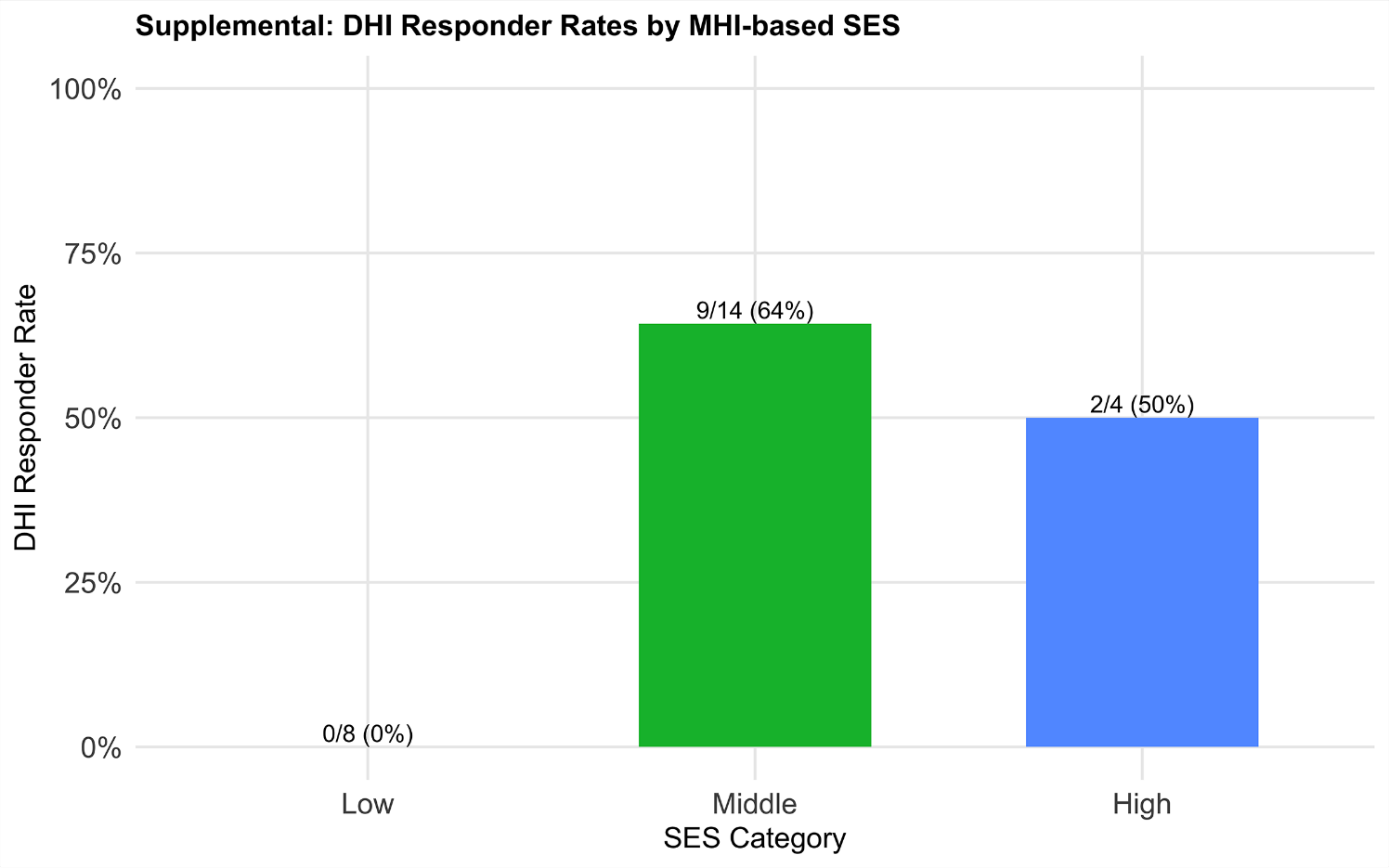


**Supplementary Figure 2. DHI Responder Rates by MHI-based SES.** Proportion of patients achieving MCID by median household income category. No low-MHI patients achieved MCID (0/8); 64.3% of middle-MHI (9/14) and 50.0% of high-MHI (2/4) patients achieved clinically meaningful improvement.

# Supplementary Tables

**Supplementary Table 1. Demographic Description of the Study Population by Vestibular Diagnosis**. Patient demographics varied by diagnosis. Mean age ranged from early 40s in BPPV to early 60s in vestibular schwannoma, with most other diagnoses clustering in the mid-to-late 50s. Most conditions showed a female predominance, except mTBI, which was predominantly male. The cohort was primarily White, with smaller proportions of Black/AA and other racial groups. The proportion of Hispanic/Latino patients varied across diagnoses, with higher representation in BPPV and mTBI and more balanced distributions in other conditions.

|  | **BPPV % (n=4)** | **mTBI % (n=7)** | **V. Migraine % (n=8)** | **V. Schwan-noma % (n=41)** | **V. Neuritis % (n=79)** | **Meniere’s disease% (n=94)** | **Other % (n=27)** | **Total Cohort (n=243)** |
| --- | --- | --- | --- | --- | --- | --- | --- | --- |
| Avg age (years) | 43 | 53 | 52 | 63 | 54 | 59 | 58 | 57 |
| **SEX** |  |  |  |  |  |  |  |  |
| *Male* | 0 | 86% (6) | 0 | 34% (14) | 25% (20) | 34% (32) | 35% (7) | 31% (75) |
| *Female* | 100% (4) | 14% (1) | 100% (8) | 66% (27) | 74% (59) | 66% (62) | 65% (13) | 69% (168) |
| **RACE** |  |  |  |  |  |  |  |  |
| *White* | 100% (4) | 86% (6) | 75% (6) | 83% (34) | 77% (61) | 86% (81) | 70% (14) | 81% (198) |
| *Black/AA* | 0 | 14% (1) | 25% (2) | 7.3% (3) | 7.5% (6) | 7% (7) | 25% (5) | 9% (22) |
| *Asian* | 0 | 0 | 0 | 0 | 0 | 1% (1) | 0 | 0.5% (1) |
| *Am. Indian/AK Native* | 0 | 0 | 0 | 0 | 2.5% (2) | 0 | 0 | 1% (2) |
| *Multiple* | 0 | 0 | 0 | 0 | 0 | 1% (1) | 0 | 0.5% (1) |
| *Unknown* | 0 | 0 | 0 | 10% (4) | 13% (10) | 4% (4) | 5% (1) | 8% (19) |
| **ETHNICITY** |  |  |  |  |  |  |  |  |
| *Hispanic/Latino* | 100% (4) | 71% (5) | 50% (4) | 37% (15) | 44% (35) | 53% (50) | 40% (8) | 47% (115) |
| *Non-Hispanic/Latino* | 0 | 29% (2) | 50% (4) | 59% (24) | 47% (37) | 39% (37) | 50% (10) | 45% (110) |
| *Unknown* | 0 | 0 | 0 | 4.9% (2) | 9% (7) | 7% (7) | 10% (2) | 8% (18) |
